# Cytochrome P450 2B6 genetic variants are associated with plasma nevirapine levels and clinical response in HIV-1 infected Kenyan women: a prospective cohort study

**DOI:** 10.1186/s12981-015-0052-0

**Published:** 2015-04-15

**Authors:** Margaret Ngwono Oluka, Faith Apolot Okalebo, Anastasia Nkatha Guantai, R Scott McClelland, Susan M Graham

**Affiliations:** Department of Pharmacology and Pharmacognosy, University of Nairobi, P.O. BOX 19498, Nairobi, 00202 Kenya; Departments of Medicine, Global Health, and Epidemiology, University of Washington, Seattle, USA and Institute of Tropical and Infectious Diseases, University of Nairobi, Nairobi, Kenya

**Keywords:** CYP2B6, Pharmacogenetics, Nevirapine, HIV infection, Antiretroviral therapy, Women

## Abstract

**Background:**

Polymorphisms in cytochrome P450 2B6 (CYP2B6) affect the steady state plasma concentration of nevirapine. CYP2B6 516G>T and 983T>C are common in African populations, but data on their influence on plasma nevirapine concentration and clinical response in African women are limited. We investigated the impact of CYP 516G>T and 983T>C on plasma nevirapine concentration and clinical outcomes in a prospective cohort study of HIV-infected Kenyan women.

**Methods:**

Study subjects were 66 HIV-1-seropositive women taking nevirapine-based antiretroviral therapy. Plasma collected at week 12 was analyzed for nevirapine concentration by high performance liquid chromatography. Baseline samples were genotyped for CYP2B6 516G>T and 983T>C single nucleotide polymorphisms by real-time polymerase chain reaction. CD4 cell count, plasma viral load, and genotypic drug resistance in plasma and genital secretions were assessed at baseline and during follow up. We evaluated the effect of each genotype on plasma nevirapine concentration at week 12 and on change in CD4 cell count at months 3, 6 and 12. Associations between plasma nevirapine concentration and clinical outcomes were analyzed by logistic or linear regression.

**Results:**

Women with CYP2B6 516TT genotype (n=9) had higher mean nevirapine plasma levels (14.33 μg/mL) compared to those with heterozygous 516GT (9.18 μg/mL; n=25) and wild- type 516GG (7.95 μg/mL; n=32) genotypes (P=0.01). Women heterozygous for the CYP2B6 983TC genotype (n=13) had higher mean nevirapine plasma levels (12.94 μg/mL), compared to women with the homozygous 983TT (8.35 μg/mL; n=53) genotype (P=0.007). In Generalized Estimating Equation analysis, plasma nevirapine levels predicted greater change in CD4 cell count after ART initiation (adjusted beta 119.4 cells/μL, 95% CI, 27.3–211.5 cells/μL, *P*=0.01). The CYP2B6 983TT genotype also predicted greater change in CD4 cell count (adjusted beta 68.6 cells/μL, 95% CI, 3.9–133.4 cells/μL, *P*=0.04). We found no associations between CYP2B6 genotypes and virologic response or toxicity.

**Conclusions:**

CYP2B6 516G>T and CYP2B6 983T>C genotypes were strongly associated with plasma nevirapine concentration, which predicted immunologic response in women on nevirapine-based antiretroviral therapy. These data support continued work on the potential utility of human genetic testing to inform nevirapine dosage optimization for individual patients.

## Background

Sub-Saharan Africa remains severely affected by HIV, accounting for 69% of people living with HIV worldwide [1]. The HIV epidemic in Kenya disproportionately affects women, with prevalence (8.0%) nearly twice that of men (4.3%) [2]. This difference is reflected in the large number of women accessing HIV care and treatment with antiretroviral drugs [3]. Over 70% of patients on first-line antiretroviral therapy (ART) in Kenya take a nevirapine-based regimen [3,4]. This is due to its relatively low cost, manageable pill burden and excellent efficacy [5]. However, the use of nevirapine is limited by a potentially fatal immune-mediated hypersensitivity reaction that manifests as hepatotoxicity, fever, and/or skin rash,[6] and by a fragile genetic barrier to the development of drug resistance [7]. Furthermore, not all patients treated with nevirapine experience optimal response. The etiology of sub-optimal response is multi-factorial and may include differences in adherence, gender, concomitant medications and genes responsible for drug metabolism [8,9].

Nevirapine undergoes oxidative metabolism primarily by cytochrome P450 3A4 (CYP3A4) and 2B6 (CYP2B6), with a minor contribution by 3A5 (CYP3A5) [10,11]. CYP2B6 metabolic activity is subject to the influence of several genetic polymorphisms, as well as strong inhibitors and inducers, contributing to highly variable plasma drug exposure [12-14]. CYP2B6 genetic polymorphisms may be more important in African populations. For example, the single nucleotide polymorphism (SNP) CYP2B6 516G>T (rs3745274), which reduces hepatic CYP2B6 protein expression and activity [15], occurs more frequently in African populations than in Caucasian and Asian populations [16-18]. Moreover, CYP2B6 983T>C (rs28399499), which is a null allele [12], is absent in Caucasians but has a prevalence of 4%–11% in African populations [18,19].

Nevirapine exhibits large inter-individual variability in its pharmacokinetics [20], contributing to variable outcomes among HIV-infected patients. Sub-therapeutic plasma concentrations are associated with treatment failure and the emergence of antiretroviral drug resistance, whereas supra-therapeutic concentrations are associated with toxicity [21]. Several studies have evaluated associations between CYP2B6 516G>T and CYP2B6 983T>C SNPs and steady-state plasma nevirapine levels [22-24]. However, prospective studies evaluating the influence of CYP2B6 polymorphisms on clinical response among HIV-infected African women taking nevirapine-based ART are lacking.

In the present study, we investigated the impact of CYP2B6 516G>T and CYP2B6 983T>C genetic variants on plasma nevirapine levels and clinical outcomes including change in CD4 cell count, plasma viral load (PVL), and the incidence of antiretroviral resistance mutations and adverse reactions.

## Results

### Baseline characteristics and plasma nevirapine levels

Sixty-nine of the 73 women screened met the inclusion criteria and were tested for CYP2B6 genotypes. Four women who had not been prescribed a nevirapine-based regimen for at least 12 weeks were excluded from analysis. Baseline characteristics of study participants at ART initiation are shown in Table 1. The median age at ART initiation was 36 (inter-quartile range [IQR] 32–40) and the median CD4 count was 126 cells/μL (IQR 79–154 cells/μL). Abnormal alanine aminotransferase (ALT) levels and skin rash were each present in 11 (16%) of the women at baseline. No baseline plasma or genital drug resistance was detected.

**Table 1:** **Baseline characteristics of study participants at initiation of nevirapine-based antiretroviral therapy**

**Characteristic**	**Median (IQR) or N (%)**
Age (yrs)	36 (32–40)
Weight (kg)	65 (54 – 70)
BMI (kg/m^2^)	25.0 (22.3 – 28.2)
CD4 cell count (cells/μL)	126 (79 – 154)
Plasma viral load (log_10_ copies/mL)	5.54 (5.17 – 5.90)
Cervical viral load (log_10_ copies/mL)	4.04 (3.42 – 4.60)
ALT levels (>ULN)	11 (15.9)
Skin rash	11 (15.9)
Plasma resistance (present)	0
Genital resistance (present)	0
ART regimen: d4T/3TC/NVP	69 (100)

BMI = body mass index, ALT = alanine aminotransferase, ULN = upper limit of normal, ART = antiretroviral therapy, NVP = nevirapine, d4T = stavudine, 3TC = lamivudine.

Sixty-six of the 69 women in this study had detectable nevirapine levels at week 12. Three women who had undetectable levels were assumed to be non-adherent and were excluded from further analysis. Steady-state plasma nevirapine levels were characterized by wide inter-individual variability, ranging from 1.68 μg/mL to 23.12 μg/mL (median 9.14 μg/mL, IQR 5.98–2.46 μg/mL). No significant correlation was found between log_10_-transformed plasma nevirapine levels and age (*P*=0.78) or body mass index (BMI, *P*=0.19).

### Frequency of CYP2B6 genotypes

For the CYP2B6 516G>T SNP, the frequency of the T variant allele was 31.2% (95% CI, 23.6%–39.6%). The number of subjects with GG, GT and TT genotypes were 35 (50.7%), 25 (36.2%), and 9 (13.0%), respectively. For the CYP2B6 983T>C SNP, the frequency of the C variant allele was 10.1% (95% CI, 5.7%–16.4%). Most individuals (55 or 79.9%) had the homozygous wild type TT genotype, whereas 14 women (20.3%) had the heterozygous mutant TC genotype and none had the homozygous mutant CC genotype. CYP2B6 516G>T polymorphism met Hardy-Weinberg (HW) equilibrium expectations (*P*=0.26). HW equilibrium could not be tested for CYP2B6 983T>C, because the homozygous mutant was not detected in this population. There was no evidence of linkage disequilibrium between rs3745274 and rs28399499 (Fisher’s exact *P*=0.45). In terms of inferred phenotypes, 26 (37.7%) individuals were predicted to be extensive metabolizers, 31 (44.9%) to be intermediate metabolizers, and 12 (17.4%) to be slow metabolizers.

### CYP2B6 516G>T and 983T>C genotypes and plasma nevirapine levels

Among the 66 women with detectable plasma nevirapine levels, CYP2B6 516G>T and 983T>C SNPs were associated with higher mean plasma nevirapine levels at week 12 (Table 2). A gene-dose effect was evident in the distribution of plasma nevirapine levels among CYP2B6 516G>T and CYP2B6 983T>C genotypes. Mean plasma nevirapine levels were higher among individuals who were homozygous for the mutation (CYP2B6 516TT, 14.33 μg/mL) and in those who were heterozygous for the mutation (CYP2B6 516GT, 9.18 μg/mL) compared to those with the wild-type (CYP2B6 516GG, 7.95 μg/mL, *P*=0.01 across groups).

**Table 2:** **CYP2B6 516G>T and 983T>C genotypes and plasma nevirapine concentrations**

**CYP2B6**	**N (%)**	**Plasma nevirapine**	**P value**
		**concentration (μg/mL),**	
		**geometric mean (95% CI)**	
CYP2B6 516G>T			
GG	32 (48.5)	7.95 (6.59-9.60)	
GT	25 (37.9)	9.18 (7.52-11.20)	
TT	9 (13.0)	14.33 (9.94-20.67)	
TOTAL	66		0.01*
CYP2B6 983T>C			
TT	53 (80.3)	8.35 (7.33-9.64)	
TC	13 (19.7)	12.94 (10.11-16.56)	
CC	0		
TOTAL	66		0.007*
Phenotypes			
Extensive metabolizers	24 (36.4)	6.92 (5.70-8.41)	
Intermediate metabolizers	30 (45.5)	9.34 (7.78-11.20)	0.02†
Slow metabolizers	12 (18.2)	14.76 (11.38-19.19)	<0.0001†
TOTAL	66		

*Comparison across groups using Stata’s *qtlsnp* command.

†ANOVA comparing either intermediate or slow metabolizers to extensive metabolizers (reference category).

**Figure 1:**
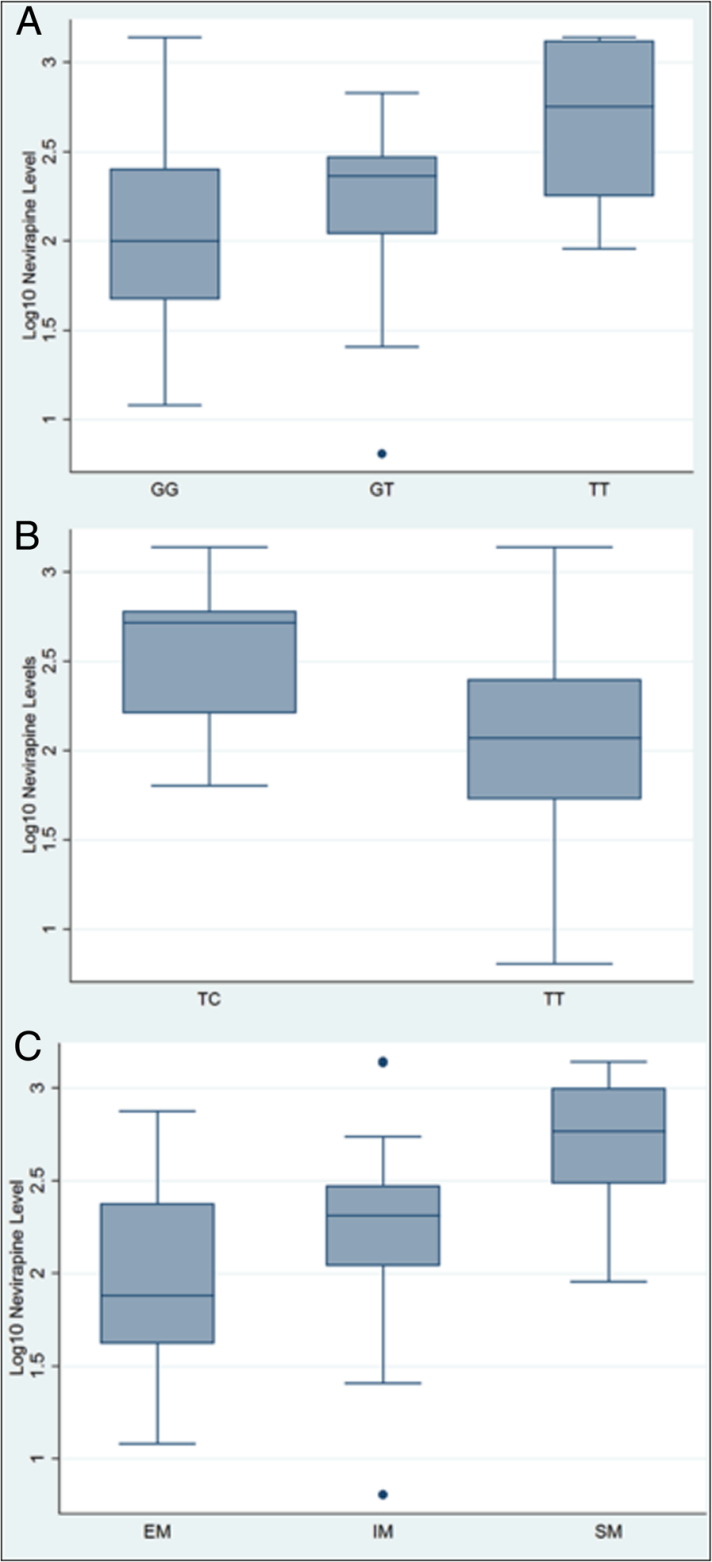
**Differences in log**_**10**_ **-transformed plasma nevirapine levels by genotype.** Panel A, Log_10_-transformed plasma nevirapine levels for each CYP2B6 516G>T genotype: GG, GT, and TT. Panel B, Log_10_-transformed plasma nevirapine levels for each CYP2B6 983T>C genotype: TC and TT. Panel C, Log_10_-transformed plasma nevirapine levels for each CYP2B6 phenotype (predicted from CYP2B6 516G>T and 983T>C genotypes). EM = extensive metabolizer, IM = intermediate metabolizer, SM = slow metabolizer.

A similar trend was observed for CYP2B6 983T>C, with mean plasma nevirapine levels being higher among individuals expressing the heterozygous genotype (CYP2B6 983TC, 12.94 μg/mL) compared to those with the homozygous wild-type (983TT, 8.35 μg/mL, *P*=0.01 across groups). With regard to the phenotypes, mean plasma nevirapine levels were higher among individuals classified as slow metabolizers (14.76 μg/mL, *P* < 0.01 compared to extensive metabolizers) and among individuals classified as intermediate metabolizers (9.34 μg/mL, *P*=0.02 compared to extensive metabolizers). The relationships between log_10_-transformed plasma nevirapine concentrations and CYP2B6 genotypes or phenotypes are summarized in Figures 1a, b and c.

### Plasma nevirapine levels predicted greater change in CD4 cell count

Higher plasma nevirapine levels were significantly correlated with greater change in CD4 cell count from baseline to month 3 (*P*=0.02) and month 6 (*P*=0.02), but not month 12 (*P*=0.07). In Generalized Estimating Equation (GEE) analysis with adjustment for baseline CD4 cell count, month since ART initiation, and time-updated adherence by pill count, plasma nevirapine levels predicted greater change in CD4 cell count after ART initiation (adjusted beta 119.4 cells/μL, 95% CI, 27.3 to 211.5 cells/μL, *P*=0.01).

### CYP2B6 983T>C genotype was associated with greater change in CD4 cell count

There was no difference in CD4 cell count at baseline between the two CYP2B6 983T>C genotype groups (mean 120 for TC, mean 123 for TT, p=0.88). Using Stata’s *qtlsnp* command, CYP2B6 983T>C genotypes were associated with mean change in CD4 cell count at month 3 (*P*=0.01), month 6 (*P*=0.02) and month 12 (*P*=0.02). In GEE analysis with adjustment for baseline CD4 cell count, month since ART initiation, and time-updated adherence by pill count, the TT genotype predicted greater change in CD4 cell count (adjusted beta 68.6 cells/μL, 95% CI, 3.9 to 133.4 cells/μL, *P*=0.04) during follow-up (Table 3). Adjustment for log_10_-transformed plasma nevirapine level diminished this effect, as shown in the last column of Table 3.

**Table 3:** **CYP2B6 983T>C genotype and change in CD4 cell count over the 12-month follow-up period**

**Variable**	**Unadjusted beta**	**P value**	Adjusted beta*	**P value**	**Adjusted beta†**	**P value**
CYP2B6 983T>C						
TT genotype	reference		reference		reference	
TC genotype	68.2 (3.3 to 133.0)	0.039	68.6 (3.9 to 133.4)	0.038	57.6 (−4.6 to 119.8)	0.069
Baseline CD4 count	0.0 (−0.4 to 0.5)	0.920	0.0 (−0.4 to 0.4)	0.831	0.1 (−0.3 to 0.5)	0.595
Months since ART initiation	3.3 (0.8 to 5.8)	0.010	3.4 (1.0 to 5.8)	0.005	3.6 (1.1 to 6.0)	0.004
Pill count adherence	0.3 (−1.6 to 2.1)	0.768	0.8 (−1.0 to 2.5)	0.384	0.7 (−1.4 to 2.8)	0.521
Log_10_-tranformed nevirapine levels	115.3 (26.6 to 204.1)	0.011			85.9 (10.6 to 161.3)	0.025

*Adjusted for all factors included in model.

†With added adjustment for log_10_ plasma nevirapine levels.

There were no differences between baseline CD4 cell count for the three CYP2B6 516G>T genotype groups (mean 127 for GG, 108 for GT, and 142 for TT, p=0.26). Interestingly, CYP2B6 516G>T genotypes had no significant effect on the mean change in CD4 cell count at month 3 (*P*=0.86), month 6 (*P*=0.61), or month 12 (*P*=0.54) in this population. This lack of effect was confirmed in a GEE model (results not shown).

### No correlation between plasma nevirapine levels and virologic response

There were no correlations between plasma nevirapine levels and change in PVL at month 3 (P=0.95), month 6 (*P*=0.53), or month 12 (*P*=0.81). In GEE analysis with adjustment for baseline PVL, month since ART initiation, and time-updated adherence by pill count, no association between plasma nevirapine levels and change in PVL was found (data not shown). *T* test comparisons showed no associations between plasma nevirapine levels and viral suppresion at month 3 (*P*=0.38), month 6 (*P*=0.59), or month 12 (*P*=0.45). In GEE analysis with adjustment for baseline PVL, month since ART initiation, and time-updated adherence by pill count, there was no association between plasma nevirapine levels and viral suppression (data not shown).

Genotypic resistance was not detected in plasma or genital secretions of study participants at baseline. During follow-up, four (5.8%) and three (4.4%) women developed genotypic resistance in plasma and in genital secretions, respectively. Plasma nevirapine levels were not associated with resistance in either plasma (odds ratio [OR] 0.55, 95% CI 0.004–81.94, *P*=0.81) or genital tract secretions (OR 3.94, 95% CI 0.006–2497.02, *P*=0.68). Adjustment for baseline CD4 cell count and adherence or for baseline PVL and adherence did not change these findings.

### Plasma nevirapine levels did not predict hepatotoxicity or skin rash

**Figure 2:**
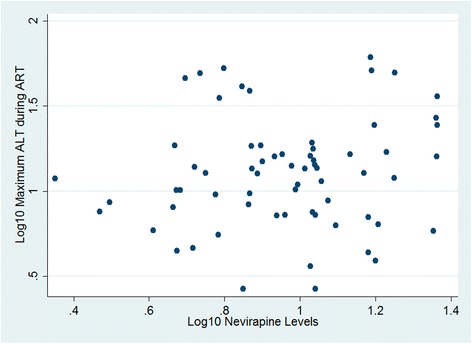
**Scatterplot of log**_**10**_ **-transformed plasma nevirapine levels and log**_**10**_ **-transformed maximum ALT levels during ART.**

Of the 69 study participants, 11 (15.9%) had abnormal ALT levels at baseline which persisted through follow-up. Of the 58 women who had normal ALT levels at baseline, 14 (24.1%) had abnormal ALT levels that developed during follow-up. Plasma nevirapine levels were not significantly associated with the development of hepatotoxicity during follow-up, despite an increased odds (OR 5.35, 95% CI 0.27–106.01, *P*=0.27). Adjustment for adherence, hepatitis B, and baseline CD4 count did not change this result (adjusted odds ratio [aOR] 2.88, 95% CI 0.13–62.95, *P*=0.50). No correlation was found between plasma nevirapine levels and maximum ALT levels recorded during ART (ρ=0.129, *P*=0.30, Figure 2). Eleven (15.9%) of the 69 study participants had a skin rash at baseline that persisted over the follow-up period. Of the 58 women with no skin rash at baseline, 27 (46.6%) developed skin rash during follow-up. Plasma nevirapine levels were not associated with a new skin rash (OR 0.36, 95% CI 0.03–3.88, *P*=0.40). Adjustment for adherence and baseline CD4 count did not change these results (aOR 0.44, 95% CI 0.03–5.97, *P*=0.54).

## Discussion

This prospective study analyzed the association between CYP2B6 516G>T and CYP2B6 983T>C genotypes, plasma nevirapine levels, and clinical response in a well-characterized cohort of HIV-1-positive Kenyan women on first-line nevirapine-based ART. The prevalence of CYP2B6 516T and CYP2B6 983C variants in this population was similar to that reported for other African populations and Kenyan ethnic groups [17,18]. We observed a significant association between both CYP2B6 516G>T and CYP2B6 983T>T genotypes and higher plasma nevirapine concentrations. In addition, plasma nevirapine levels significantly predicted change in CD4 cell counts after ART initiation. Furthermore, CYP2B6 983TC heterozygosity was associated with greater increase in CD4 cell count over the 12-month follow-up period.

CYP2B6 983T>C occurs predominantly in African subjects, with allele frequencies of 4% to 11% [19,20]. Homozygosity for the mutant allele CYP2B6 983 CC was not detected in this population, in concurrence with a previous study [25]. However, heterozygosity for the mutation CYP2B6 983TC was associated with higher plasma nevirapine levels in our study, with heterozygous TC individuals having levels 55% higher than those with the wild type TT. This effect is noteworthy for its magnitude, especially when compared to the smaller effect observed for CYP2B6 516GT (16%). The CYP2B6 983T>C (rs28399499) SNP results in the variant protein CYP2B6*18 with an I328T as the only amino acid change. Its expression *in vitro* results in no detectable protein or activity [26], and it has been designated as a null allele [12]. This null status could explain the greater impact of CYP2B6 983TC compared to the CYP2B6 516GT. In agreement with our results, other studies have reported that CYP2B6 983TC heterozygosity leads to reduction in nevirapine clearance [27,28].

We also made an observation that heterozygosity for CYP2B6 983TC was significantly associated with greater increase in CD4 cell count during follow-up. This observation could be attributed to the fact that CYP2B6 983TC was associated with higher plasma nevirapine levels, which in turn were associated with greater increase in CD4 cell count, particularly in the early months of ART. In fact, from our multivariable analysis, the addition of plasma nevirapine levels to the model weakened the association between genotype and change in CD4 cell count, providing evidence of at least partial mediation of this association by nevirapine levels.

Previous studies evaluating the impact of CYP2B6 genotypes on clinical outcomes have reported varied results. A study by Haas et al. [29] reported no association between CYP2B6 516G>T genotypes and change in CD4 cell count, in concurrence with our findings. However, in a HIV-1-positive pediatric cohort, CYP2B6 516TT genotype was associated with greater increase in CD4 cell count percentage at week 12, compared to those with the GT and GG genotypes (9.0% vs. 5.0% vs. 3.2% increases, respectively) [30]. More recent studies report a significant association between the CYP2B6 516 T allele, UGT2B7*2, higher efavirenz plasma levels and change in CD4 cell count in HIV-1 positive African patients in Tanzania and Ethiopia [31]. These studies did not include testing for CYP2B6 983T>C. Hence, our study is the first to report a significant association between CYP2B6 983TC heterozygosity and greater increase in CD4 cell count in an African population.

It is unclear why nevirapine concentrations would influence CD4 cell recovery but not have a detectable effect on viral load reduction or suppression. At least one study of patients with virologic suppression on nevirapine has highlighted nevirapine’s ability to penetrate reservoir sites and achieve high intracellular concentrations, leading to extremely low levels of viral replication [32]. It is possible that penetration into reservoir sites varied by genotype in this study, leading to an impact on CD4 T cell recovery that was independent of the effect on plasma viral load. Alternatively, because most women achieved virologic suppression and there were no differences in baseline plasma viral load prior to ART initiation, there may have been a floor effect on change in plasma viral load that reduced our power to detect differences by genotype. In contrast, our study participants initiated therapy with very low CD4 counts which increased during ART and reached no ceiling during the 12 months of follow-up. We may, therefore, have had more power to detect differences in CD4 cell recovery. A third explanation is that some other genetic trait linked to the CYP2B6 983T>C phenotype somehow influences CD4 T cell recovery. This is a potential area of investigation, should our results be confirmed in other studies.

A major strength of our study was its design as a prospective study in a well-characterized cohort in which clinical outcomes were carefully measured and recorded. Our study also had a number of limitations. First, sub-optimal adherence could have contributed to the observed wide inter-patient variability in plasma nevirapine levels. Second, plasma nevirapine levels could also be under the influence of other drug metabolism and membrane transporter genes which were not studied in our cohort. Third, we do not have information on nutritional status, herbal remedies, or medications obtained outside the research clinic that may have influenced immune response. Fourth, our sample size and duration of follow-up were limited, and we may have failed to detect smaller differences in some outcomes. Despite these limitations, we were able to identify several clinical correlates that were associated with participant genotypes.

## Conclusions

In conclusion, we have found in this study that CYP2B6 516G>T and 983T>C genotypes are associated with higher plasma nevirapine exposure in HIV-1 infected Kenyan women, and that higher plasma nevirapine levels and the CYP2B6 983TC genotype were associated with better immunologic response. In this study, neither plasma nevirapine levels nor CYP2B6 genotypes were significantly associated with toxicity or with the emergence of plasma or genital resistance. However, our follow-up time was limited and the incidence of both toxicity and drug resistance was fairly low. We recommend further studies to confirm the role of CYP2B6 genotypes and other relevant genes in the pharmacokinetics and clinical response to nevirapine in HIV-1-positive African women.

## Methods

### Study population and design

The study was nested within an ongoing prospective cohort study investigating the impact of ART on HIV-1 infectivity in female sex workers in Mombasa, Kenya [33,34]. At the time of this study, ART was initiated for women with CD4 counts ≤200 cells/μL or an AIDS defining illness [35]. Pregnant women were excluded and instead referred to programs offering ART for the prevention of mother to child transmission. Women requiring rifampicin-containing treatment for active tuberculosis were prescribed efavirenz or a triple non-nucleoside reverse transcriptase inhibitor regimen, according to WHO guidance at the time [35]. All women were prescribed co-trimoxazole prophylaxis, unless contraindicated.

The standard ART regimen included nevirapine 200 mg daily for 14 days, then 200 mg twice daily [35]. The NRTI backbone included stavudine or zidovudine plus lamivudine at standard doses [35]. Adherence was promoted by directly observed administration of one of the two daily doses to the women on weekdays during their first month of ART. Pill box organizers and a monthly support group were also used to enhance adherence, which was monitored at each visit by pill count and a validated visual analogue scale [36]. Fluconazole, which can impact nevirapine levels, was prescribed for only one woman in this study, starting at the month 6 visit.

Clinical response to treatment was monitored, including self-reported symptoms and physical examination (including rash). Laboratory testing, including regular testing of CD4 cell count, hemoglobin, ALT, creatinine, and plasma and genital viral load, were performed on stored samples from baseline, month 3, month 6, and month 12 [33,34]. Samples with detectable HIV-1 ribonucleic acid (RNA) after treatment initiation were evaluated for antiretroviral resistance mutations.

All HIV-1-seropositive women who initiated nevirapine-based ART between May 2005 and September 2007 and had stored peripheral blood mononuclear cells (PBMCs) and plasma collected at week 12 of ART were included in this study. Women in the parent ART cohort gave consent at enrollment for long-term storage of samples and for additional studies including genetic testing. The present study was approved as a protocol modification by the University of Washington Human Subjects Research Committee and Kenya Medical Research Institute’s Ethics and Research Committee.

### Determination of plasma nevirapine levels

Blood plasma collected 10–14 weeks after ART initiation was used for the determination of plasma nevirapine concentrations. Blood collection was not timed in relation to medication dosing, since this pharmacological study was a secondary use of stored samples and data. However, most women attended clinic in the morning, and are likely to have been sampled between 2 and 4 hours after pill ingestion, at an optimal sampling time for nevirapine [37]. Plasma nevirapine concentrations were quantified using a reversed phase high performance liquid chromatographic method adapted from the method of Minzi and Ngaimisi [38]. Calibration curves obtained for nevirapine-spiked plasma samples were linear, with correlation coefficients (r^2^) in the range of 0.993–0.996 and a limit of quantitation of 0.86 μg/mL. The intra-day and inter-day precision (CV%) ranged from 6.08–7.96 and 4.72–7.32, respectively. Intra-day and inter-day accuracy (RD%) ranged from 3.47–5.51 and 2.00–8.10, respectively. The mean recovery for low, medium and high concentrations of nevirapine was in the range of 96–110%.

### DNA preparation and genotyping procedures

DNA was extracted from PBMCs using the QIAamp DNA blood mini-kit (Qiagen GmbH, Hilden, Germany) according to the manufacturer’s protocol. The extracted DNA was quantified using a UV spectrophotometer ND-1000 (NanoDrop Technologies, Wilmington, DE, USA) at 260 nm and normalized to a concentration of 20 ng/mL. Genotyping was carried out on an ABI 7500 Fast Sequence Detection System (Applied Biosystems, Foster City, CA, USA). SNPs were analyzed using the validated Taqman Genotyping Assays purchased from Applied Biosystems for CYP2B6 516G>T (rs3745274; assay ID C_7817765_60) and CYP2B6 983T>C (rs28399499; assay ID C_60732328_20), according to the manufacturer’s instructions.

### Definitions

CYP2B6 516G>T and 983T>C genotypes were described as homozygous wild type for the G or T alleles (516GG or 983TT), heterozygous (516GT or 983TC), or homozygous mutated (516TT, 983CC). CYP2B6 metabolic phenotypes were inferred from composite CYP2B6 genotypes as previously described [22]. Those with no variant alleles at either positions (516GG or 983TT) were classified as “extensive metabolizers.” The term “intermediate metabolizers” described those with a single variant allele at either position, but not both. The term “slow metabolizers” described those with two variant alleles (516TT, 983CC, or 516GT with 983TC).

Changes in CD4 cell count and PVL were assessed at months 3, 6, and 12. Viral suppression was defined as <100 copies HIV-1 RNA per mL of plasma. Hepatotoxicity potentially attributable to ART was defined as a normal ALT at baseline with an elevation above the upper limit of normal during treatment. A possible ART-related skin rash was defined as no rash at baseline followed by rash during treatment. Plasma or genital drug resistance was defined as identification of drug resistance mutations in plasma or genital secretions before or during treatment.

### Data analysis

The prevalence of each genetic variant was estimated from the total number of copies of individual alleles divided by the number of all alleles in the study population. Deviation of genotypes from HW equilibrium expectation was estimated using Stata’s *hwsnp* command, which calculates an exact HW significance probability. Prevalence estimates were reported with exact binomial confidence intervals. Steady-state plasma nevirapine levels were tested for normality by the Shapiro-Wilk test, and subsequently log_10_-transformed due to lack of normality. All averages are hence presented as the geometric mean. Women with undetectable levels of nevirapine were excluded from analysis, because it was assumed that they were non-adherent to treatment.

One-way analysis of variance was used to determine whether there were significant differences in continuous variables across genotype or phenotypes groups. The effects of each genotype or phenotype on plasma nevirapine levels at week 12 and on change in CD4 cell count at month 3, 6 and 12 were evaluated using Stata’s *qtlsnp* command, which assumes a co-dominant genetic model and tests for an additive effect, a dominant effect and that both effects are equal to zero (this last comparison is equivalent to comparing means across the three possible genotypes and phenotypes). We also modeled the relationship between plasma nevirapine levels and post-ART outcomes including change in CD4 count, plasma viral load, and viral suppression using GEE models with a logit or identify link, independent correlation matrix, and robust standard error estimates. We adjusted these models for month since ART initiation, time-updated adherence by pill count, and either baseline CD4 count or plasma viral load, as appropriate. To determine whether plasma nevirapine levels acted as a mediator in the association between CYP2B6 983T>C genotype and change in CD4 cell count, the effect of adding log_10_-transformed plasma nevirapine levels to multivariable modeling was also evaluated by GEE analysis.

The correlation between log_10_-transformed plasma nevirapine levels and age, BMI, change in CD4 cell count, and change in log_10_-transformed PVL at months 3, 6 and 12 was evaluated with the Pearson’s test for correlation. An independent *t* test was used to compare log_10_-transformed nevirapine levels in women with suppressed versus unsuppressed PVL. Logistic regression was used to test for associations between log_10_-transformed plasma nevirapine levels and the development of ALT elevation, skin rash, or plasma or genital drug resistance after treatment initiation. The modeled binary outcomes were adjusted for potential confounding factors appropriate to the outcome being evaluated. In the analysis of hepatotoxicity, the presence of hepatitis B infection was included in the model as a potential effect modifier. Data were analyzed using Stata version 11.2 (Stata Corp, College Station, Texas, USA).

## Abbreviations

ALT: Alanine aminotransferase
AOR: Adjusted odds ratio
ART: Antiretroviral therapy
BMI: Body mass index
CI: Confidence intervals
CYP2B6: Cytochrome P450 2B6
CYP3A4: Cytochrome P450 3A4
CYP3A5: Cytochrome P450 3A5
GEE: Generalized estimating equation
HW: Hardy-Weinberg
IQR: Inter-quartile range
OR: Odds ratio
PBMC: Peripheral blood mononuclear cells
PVL: Plasma viral load
RNA: Ribonucleic acid
SNP: Single nucleotide polymorphism
